# Stromal-epithelial responses to fractionated radiotherapy in a breast cancer microenvironment

**DOI:** 10.1186/s12935-015-0218-9

**Published:** 2015-06-27

**Authors:** Muqeem A. Qayyum, Jin Tae Kwak, Michael F. Insana

**Affiliations:** Department of Bioengineering, University of Illinois at Urbana-Champaign, 405 North Mathews Avenue, Urbana, IL 61801 USA; Department of Nuclear, Plasma, and Radiological Engineering, University of Illinois at Urbana-Champaign, 405 North Mathews Avenue, Urbana, IL 61801 USA; Department of Electrical and Computer Engineering, University of Illinois at Urbana-Champaign, 405 North Mathews Avenue, Urbana, IL 61801 USA; Beckman Institute for Advanced Science & Technology, University of Illinois at Urbana-Champaign, 405 North Mathews Avenue, Urbana, IL 61801 USA

**Keywords:** Stromal-epithelial, Metabolic active, Fractionation, Reactive stroma

## Abstract

**Background:**

The stromal-epithelial-cell interactions that are responsible for directing normal breast-tissue development and maintenance play a central role in the progression of breast cancer. In the present study, we continued our development of three-dimensional (3-D) cell co-cultures used to study cancerous mammary cell responses to fractionated radiotherapy. In particular, we focused on the role of the reactive stroma in determining the therapeutic ratio for post-surgical treatment.

**Methods:**

Cancerous human mammary epithelial cells (MRC-7) were cultured in a 3-D collagen matrix with human fibroblasts (MRC-5) stimulated by various concentrations of transforming growth factor beta 1 (TGF-β1). These culture samples were designed to model the post-lumpectomy mammary stroma in the presence of residual cancer cells. We tracked over time the changes in medium stiffness, fibroblast-cell activation (MRC-5 converted to cancer activated fibroblasts (CAFs)), and proliferation of both cell types under a variety of fractionated radiotherapy protocols. Samples were exposed to 6 MV X-rays from a linear accelerator in daily fraction sizes of 90, 180 and 360 cGy over five days in a manner consistent with irradiation exposure during radiotherapy.

**Results:**

We found in fractionation studies with MRC-5 fibroblasts and CAFs that higher doses per fraction may be more effective early on in deactivating cancer-harboring cellular environments. Higher-dose fraction schemes inhibit contractility in CAFs and prevent differentiation of fibroblasts, thereby metabolically uncoupling tumor cells from their surrounding stroma. However, higher dose fraction appears to increase ECM stiffening.

**Conclusions:**

The findings suggest that dose escalation to the region with residual disease can deactivate the reactive stroma, thus minimizing the cancer promoting features of the cellular environment. Large-fraction irradiation may be used to sterilize residual tumor cells and inhibit activation of intracellular transduction pathways that are promoted during the post-surgical wound-healing period. The higher dose fractions may slow wound healing and increase ECM stiffening that could stimulate proliferation of surviving cancer cells.

## Background

There are several significant challenges to the successful treatment of breast cancer in a heterogeneous patient population. One primary challenge is the effective application of adjuvant therapies to individuals; for example, to determine the best fractionation schedules for minimizing cancer recurrence using photon radiotherapy in post-lumpectomy patients. There is now strong evidence that stromal-epithelial cell signaling during wound healing plays an important role in answering that question [1]. Heterotypic cell signaling is known to modulate the formation and growth of tumors, and hence it can influence the radiosensitivity of residual cancer cells within the reactive stroma microenvironment.

Properties of mesenchymal cells, the extracellular matrix (ECM), and other insoluble proteins surrounding cancer cells facilitate the production and storage of growth factors that modulate the intensity of essential cell signals. The stiffness of the ECM also directly influences cell behavior during tumor development via the ability of cells to transduce adhesion forces into protein expression [2]. While it is difficult to untangle how these myriad coupled signals are combined to promote or inhibit tumor growth, obtaining greater insights into this complex process can help guide the design of safer and more effective cancer treatments.

It is especially difficult to follow, *in vivo*, the multifactorial influences of cellular microenvironmental signaling during a course of radiotherapy. So we abstracted key elements of the post-surgical wound-healing microenvironment in standard three dimensional (3-D) cell cultures to isolate the most important influences. In this report, we co-cultured cancerous epithelial cells (cancer cells) with fibroblast cells in which some of the fibroblasts were prompted by growth factors to differentiate into cancer-associated fibroblasts (CAFs). CAF phenotype was measured through the expression of α–SMA using immunofluorescence staining. Under these conditions fibroblasts are generally called carcinoma associated fibroblasts (CAFs) [3]. These co-culture samples were designed to simulate features of the reactive stroma within a surgical wound containing residual tumor cells. The resulting data provides insights into the effects of different microenvironmental conditions on the therapeutic ratio of fractionated radiotherapy.

The co-cultures used in this report expand the number of cellular influences that we assembled during a previous study of the reactive stroma [4]. Culturing cancer cells with reactive stromal cells has allowed us to observe the effects of heterotypic interactions on the therapeutic ratio during treatment. The therapeutic ratio is the kill rate of targeted cancer cells relative to that of non-targeted cells for a given fractionation schedule. Of course, co-cultures cannot include the entire range of *in vivo* influences, especially important systemic factors that mediate cell responses to radiotherapy. The input parameters of this physical model are the initial numbers of cells, the concentration of applied growth factors, and fractionation parameters (timing and dose per fraction). The output parameters of the model are the time-varying numbers of each cell type (proliferation rates), fraction of differentiated fibroblasts (fibroblast activation rate), and 3-D collagen-matrix stiffness that describes the combined effects of CAF contraction and radiation-induced collagen-matrix cross linking. Study results may ultimately guide the adjustment of fractionation parameters in patient treatments by providing criteria for balancing cellular radiosensitivity and sub-lethal damage repair rate while maximizing patient tolerance.

To our knowledge, no systematic investigation involving co-culture models of residual disease in the post-surgical environment has been conducted to observe cancer cell behavior for different radiation fractionation schedules. Studying how radiotherapy may influence the release of stimulatory factors during wound healing might also lead to the identification of new biomarkers for monitoring treatment responses. We hypothesize that 3-D cell co-cultures, with an appropriate blend of environmental influences, can provide predicative models for such investigations, where the goal is to deactivate cancer cells as well as the reactive stroma so the latter does not act as a promoter of the former. It seems possible to design a fractionation prescription that is based on stromal biomarkers and that eliminates all residual disease.

## Results and discussion

### Calibration of MTT absorbance measurements

**Fig. 1:**
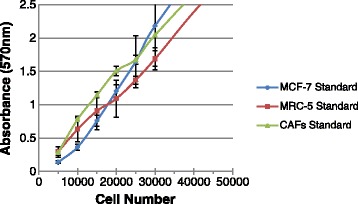
MTT standard curves. Cells curves for monoculture cells plated in a serial dilution ranging from 5000 to 120,000 cells/μl. Samples were incubated for 4 h with MTT as described in Materials and Methods. At the end of the MTT-incubation period, the formazan crystals were dissolved in DMSO. During the experiment the formazan/dimethyl sulphoxide solution was kept in the dark at room temperature. In the range 10,000 and 30,000 cells/μl, the data indicate a linear relationship between absorbance signal and cell number. Error bars indicate one standard error of the mean (SEM)

Three cell types (MCF-7, MRC-5, and MRC-5 activated by TGF-β1 to become CAF) underwent MTT assay analysis. All three cell types were plated into 96 well plates individually in serial dilutions of cell density ranging from 5000 – 120,000 cells/μl. To calibrate our system, we first developed standard curves for each cell type, as shown in Fig. 1. This was done by plating the full range of cell densities in a 96-well plate and allowing time for cell attachment but minimal propagation. We then added the MTT assay reagent to measure the exact absorbance level of each well. The assay was allowed to incubate for 4 h, before the medium was removed and the dye was dissolved with 100 μl of DMSO. The optical density was measured at 570 nm using a spectrophotometer to estimate absorbance.

The data in Fig. 1 indicate a logistic-curve relationship between the absorbance signal and cell number. The appearance of the curve is explained from known cell behaviors at three density ranges. At the lowest cell densities (<10,000 cells/μl), there is less direct contact among cells and hence less of the paracrine signaling that enables normal metabolism [5]. Here normal metabolic activity is suppressed. In the cell-density range of 10,000 - 30,000 cells/μl, MTT absorbance increases linearly with cell density, which is expected if metabolic activity for all cells is roughly equal. Cells are dense enough for the natural paracrine signaling that enables normal metabolic activity. When cell density is too high, cells quickly deplete vital nutrients from the medium, which suppresses the average metabolism per cell. Also at high densities, optical absorbance can be above the operating range of 4 units where the detector responds nonlinearly. Thus, in the range from 10,000 to 30,000 cells/μl, absorbance increased approximately linearly with cell density.

**Fig. 2:**
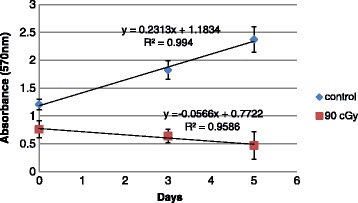
MCF-7 metabolic activity. The MTT assay for MCF-7 cells under irradiated and non-irradiated conditions for 2D monoculture. The two data sets were fit to a straight line using linear regression. Slopes indicate changes in the metabolism of cells in the sample over time. A positive slope occurs during cell proliferation and a negative slope occurs as the cancer cells respond to daily doses of radiation. Error bars indicate one standard error of the mean (SEM)

Once the linear range was established from the standard curves in Fig. 1, we chose a fixed cell density of 20,000 cells/μl to observe the metabolic changes in samples over 5 days. Unlike samples used in the standard curve development, these cells proliferated during the 5-day period. The absorbance values on days 0, 3 and 5 were measured and absorbance slope was used as the relative metabolic activity. Figure 2 shows plots of absorbance for non-irradiated (control) MCF-7 cell samples and those irradiated at the 90 cGy fraction dose conditions.

### Absorbance as a measure of cell proliferation

**Fig. 3:**
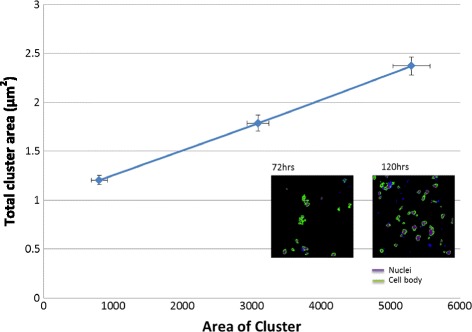
Relationship between metabolic activity and surface area in MCF-7 cells. Absorbance from an MTT assay for a sample with an initial plating density of 20,000 cells/μl. Absorbance was measured on days 0, 3, and 5 days for MCF-7 cells versus the area occupied by these cells at the same times using IF stained images (INSET). Error bars indicate one standard error of the mean (SEM)

Within the linear portion of the absorbance curve, absorbance measurements indicate relative cell number. To establish the necessary relationship between MTT absorbance and cell number, we also manually counted cells in confocal microscopy images of α-SMA, IF stained cell samples. Clustering of MCF-7 cells made accurate cell counting difficult. Instead, we measured the fractional area occupied by cell clusters during the 5 day period and plotted that area measurement versus absorbance. We found the linear functional relationship shown in Fig. 3, where the correlation coefficient for the linear regression R^2^ = 0.9999. Assuming non-overlapping cells, constant metabolic activity per cell, and absorbance in the linear range of measurements, we established that absorbance is a proportional to cell number. Hence absorbance rates indicate relative cell proliferation rates.

### Metabolic activity and cell type

Although the standard calibration curves for each cell type in Fig. 1 are each linear over the same cell density range, the slopes in that range are different indicating different metabolic rates for each cell type. Specifically, different cell types produce formazan at different rates, which varies the slope of the absorbance curve. We notice in particular that CAF appear to exhibit a higher metabolic rate than non-activated MRC-5 fibroblasts for the same number of cells. Also, unlike measurements using IF stained images, the MTT assay naturally distinguishes between viable and dead cells.

### Metabolic activity and IR fractionation

**Fig. 4:**
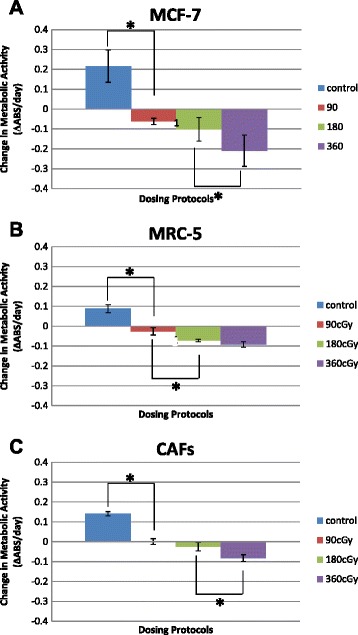
Change in metabolic acidity for dosing protocols. Metabolic activity of MCF-7, MRC-5 and CAF cells measured over 5 days at different dose fractions in 2D culture. **a** MCF-7 cancer cell metabolic activity indicating radiation sensitivity. **b** MRC-5 metabolic activity and **c** CAFs metabolic activity showing more radio-resistant behavior than MRC-5 and MCF-7 cells. Error bars indicate one SEM. *indicates statistical significance at the *p* < 0.05 level based on ANOVA analysis

Figure 4 displays the change in metabolism measured over five days (metabolic rate) for the three cell types cultured independently. Samples were exposed at one of three different dose fractions, and there were non-irradiated control samples in each case. A rate listed as zero indicates no change in cellular metabolism of a sample from the initial value measured on day 0. Measurements on control samples for all three cell types (Fig. 4a-c) are positive, indicating an increase in overall metabolism for cells in these samples over five days. We interpret a positive rate as cell proliferation for the reasons discussed above.

The MCF-7 (cancerous epithelial) cells have the highest control (non-irradiated) metabolism. They also have the greatest sensitivity to radiation. Specifically, higher dose fractions induce a greater reduction in the metabolic activity of a sample compared with the other two cell types. The reduction can be due to combinations of cell death and reduced metabolic activity in surviving cells, but we assume the change is dominated by cell death. We see in Fig. 4a that the surviving fraction of cells in irradiated samples was significantly lower than that in the untreated controls, indicating, as expected, that of the three types of cells MCF-7 are most sensitive to radiation (overall *p* <0.05).

We also studied MRC-5 fibroblasts (Fig. 4b) under exogenous growth-factor free conditions and CAFs (Fig. 4c) that were activated by exposing MRC-5 s to TGF-β1. 10 ng/ml TGF-β1(Sigma) in 0.5 % FBS/MEME. TGF-β1 was applied in a single treatment application prior to the irradiation. We experimented with different TGF-β1 concentrations in the range of 0.10–10 ng/ml and measured the cell activation that resulted. Normally MRC-5 fibroblasts are more radio-resistant than MCF-7 cancer cells. We found that CAFs are the most radio-resistant of the three types.

We find that sample absorbance (and hence cell number) decreases with radiation dose just as others reported [6]. If decisions on fractionation strategy are based on the survival of cancer cells alone, the results of Fig. 4a would suffice. The standard 180 cGy fraction might be selected to save the patient some extra risk. However, since the microenvironment also consists of CAF whose population we also wish to minimize, Fig. 4c shows that a standard fraction gives a relatively small CAF response, and that the higher 360 cGy fraction would be much more effective at reducing the CAF and cancer cell populations provided that dose can be tolerated by the patient. Eliminating the CAF population removes an important proliferative factor if there are residual cancer cells present in the stroma tissue of the surgical wound. However, activated fibroblasts are also important for the wound-healing process itself, and therefore a higher fraction may slow the healing process. Large fraction sizes have been proposed as a result of the START trials [7] that favor use of hypofractionated (i.e., larger fraction size) breast irradiation because it provides excellent long-term local control and limited late morbidity as well as benefits of convenience and cost.

### Stromal fibroblasts stimulate cancer cell growth

To characterize the behavior of cells in 3-D collagen co-culture samples, MCF-7 cells were embedded in collagen gels, with or without the addition of MRC-5 fibroblasts. Based on preliminary studies, a 3:1 ratio was chosen of MRC-5 to MCF-7 cells, respectively, to be used throughout the investigation. Since the MTT assay provides information on total metabolic activity for a sample, we detected the area of MCF-7 cells in a sample through microscopy using IF staining in which MCF-7 and MRC-5 fibroblast cells can be easily identified, counted, and measured.

**Fig. 5:**
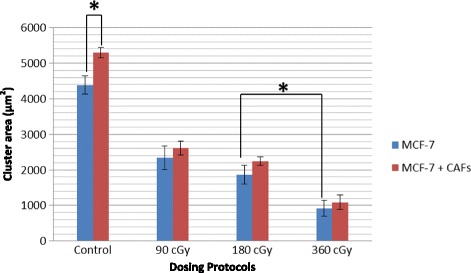
MCF-7 cluster area at 5 days. MCF-7 cell cluster areas were measured after 5-days for control and irradiated samples and for mono- and co-cultures. The presence of MRC-5 cells in the co-cultures induced MCF-7 cell growth, resulting in large, irregular, and partially branched epithelial cell clusters. *indicates statistical significance at the *p* < 0.05 level based on ANOVA analysis

Mixing MRC-5 fibroblasts with MCF-7 cancer cells in co-cultures further stimulated MCF-7 cell growth, as expected, resulting in larger and more abundant MCF-7 cell clusters (Fig. 5). MCF-7 cell clusters composed of one cell to more than a hundred cells were included in the analysis. The increase in MCF-7 proliferation was significant from day 3 onward (data not shown).

### Co-culture responses to IR

Co-culture samples were exposed to 5 sequential daily fractions of 90, 180 or 360 cGy/fraction, for a total dose of 450, 900 and 1800 cGy. The numbers of cells on day 5 that resulted from proliferation and radiation treatments are illustrated in Fig. 5. MCF-7 cluster area was reduced monotonically with fraction size. Exposed to the same amounts of radiation, the average MCF-7 area was larger in co-cultures than monocultures, though the difference was not significant at *p* = 0.05. The largest fraction size of 360 cGy produced disproportionately more damage than smaller fraction sizes (*p* < 0.05) in MCF-7 areas (Fig. 5). We believe these finds may demonstrate the importance of regulating cancer cell survival and proliferation during treatments.

### Fibroblast ability to contract after IR

**Fig. 6:**
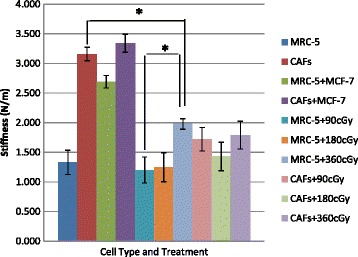
Stiffness. Measurements of collagen matrix stiffness were made after three days of culturing for samples formed with fibroblast cells in co- and monocultures. This samples that were stimulated with TFG-β1 and/or with radiation are indicated. Irradiated samples were irradiated once each day at the daily dose indicated for three days. Each bar indicates the average stiffness from one measurement taken on each of 6 samples (mean ± 1 SE). *indicates statistical significance at the *p* < 0.05 level based on ANOVA analysis

We went on to investigate another functional status of irradiated cells – collagen matrix stiffness – which has been shown to be a significant factor influencing tumor cell invasiveness and angiogenesis [1,2]. MRC-5 fibroblasts in collagen were found in our previous study to stiffen the collagen matrix somewhat. However, if we add TGF-β1 that is known to transform MRC-5s into CAFs, the collagen matrix stiffness more than doubled the control value as the CAFs contract (Fig. 6). In one set of samples, growth factors were not added to co-cultures. Nevertheless, growth factors are produced by cancer cells, and so it is not surprising that we see nearly the same stiffening response as in equivalent co-culture samples with growth factors added. Matrix stiffness values were largest, in the range of 2.5 to 3 N/m for control samples when TGF β1 is present with fibroblasts.

Irradiating MRC-5 cells not exposed to growth factor, we found matrix stiffness increased with dose, we believe this may be due to production of reactive oxygen species (ROS) as a result of the absorbed radiation induced cross linking of the collagen matrix [8,9]. This mechanism involves the activation of specific proteolytic enzymes from inactive proenzymes and is most likely dependent on the presence of reactive oxygen species (ROS). Scavengers of radiation-induced free radicals or ROS, such as amifostine, can inhibit ROS-induced activation of LTGFβ as well as diminish the plasma level of TGF-β1 [10].

Irradiating co-cultures with TGF β1 at 90 cGy reduced the stiffness of the collagen relative to the non-irradiated samples but not as much as the reduction seen at the 180 cGy fraction. We believe the increase in stiffness with the larger 360 cGy fraction indicates that ROS-induced collagen cross linking is greater than CAF inactivation. Overall, these results indicate that while CAF appear to survive high radiation doses, their contraction capacity may be considerably compromised.

Our data show that ionizing radiation generally reduces factors in the reactive stroma that contribute to cancer cell proliferation. However excessive radiation can stiffen the ECM that can promote proliferation of surviving cancer cells.

## Conclusions

We see our investigation as a first step in a new approach to radiotherapy planning involving the radiobiology of cell cultures. In the past, radiobiology looked at responses of cell types under isolated conditions. We have begun to generalize these experimental conditions to include 3-D co-culture models to evaluate the dose fractionation schemes. However our studies have examined short-term effects, essentially those after 1 week of treatment, when clinical goals are met only if disease fails to recur after years, e.g., 5-years survival.

We maintain the radiobiological focus on cancer cell responses but only for cells placed in a more natural microenvironment, which acknowledges that the reactive stroma is an important influence on both tumor recurrence and post-lumpectomy healing. So the feature space of analysis is expanded to include fibroblast activation, ECM stiffness, and CAF influences as well as cancer-cell proliferation since each are important parameters influencing the survival and phenotype of residual disease.

Myofibroblasts or CAFs have been found in a variety of normal tissues and various pathological situations, such as wound healing, fibromatosis, and stromal reaction to epithelial tumors [11]. We found in fractionation studies with MRC-5 fibroblasts and CAFs that higher doses per fraction may be more effective early on in deactivating cancer-harboring environments. Higher fraction schemes inhibit contractility in CAFs and prevent differentiation of fibroblasts, thereby metabolically uncoupling tumor cells from their surrounding stroma. Yet, over a time period longer than 5 days, the higher dose fractions in patients may slow wound healing, and the increase in ECM stiffening could stimulate any surviving cancer cells.

The conclusion that MRC-5 fibroblasts definitively modulated the response of MCF-7 cells to radiation relies on two main considerations. First, using the MTT assay on different cell types, CAFs were found to be most resistant to radiation in terms of metabolic activity, i.e., for all fractionation schemes on days 3 and 5 (Fig. 3). The observation that CAFs were more radio-resistant and MCF-7 cells more radiosensitive suggested that the target of interest should also include the CAFs cells. These observations emphasize the metabolic cooperation between residual cancer cells and newly formed CAFs. Second, we saw that 3-D co-cultures of MCF-7 and MRC-5 cells produced significantly larger cancer-cell clusters than that in samples containing MCF-7 cells alone (Fig. 4). Taken together, these results suggest that fibroblasts and cancer cells in co-culture become metabolically coupled.

Many investigators have stressed the unique synergistic characteristics between cancerous mammary epithelia and CAF [12, 13]. In essence, together with the ECM, these are the main components of stromatogenesis; i.e., the process of new stroma formation occurring concurrently with tumor cell invasion and angiogenesis. The newly formed stroma not only provides tumor cells with a substrate suitable for tumor cell growth and proliferation but also offers them metabolic support essential for cancer cell survival. These findings direct attention to the stromal components along with the tumor, which might give rise to an improved therapeutic ratio.

Tumors are characterized by ECM remodeling and stiffening [14]. Our experimental results both improve our basic understanding of mechanobiology of the microenvironment, metabolic activity, as well as identify relevant therapeutic responses. Many investigators have found breast cancer tissue to be as sensitive to fraction size as dose-limiting healthy tissues [7, 15, 16]. Fibroblasts, CAFs and the factors they produce are important targets for RT and prove to be useful prognostic markers. These findings confirm that radiotherapy schedules can be evaluated through metabolic activity, stiffness and differentiation changes.

Expanding the study to include essential elements of the tumor microenvironment provides new insights into long term sustainability of tumor regeneration. Evaluation of alternative fractionation techniques, e.g., hypofractionation [7], may result in a useful alternative to standard breast cancer RT for some patients. There is value in a technique which reduces the treatment time while maintaining cosmetic and functional control, if adjusting fraction size from standard protocols does not adversely affect stiffness or differentiation changes.

## Methods

### Cell lines and co-cultures

The human mammary carcinoma cell line MCF-7 and the human lung fibroblast cell line MRC-5 were obtained from the ATCC (Rockville, MD, USA). Collagen type I stock solution (10 mg/ml, rat tail) were purchased from BD Biosciences (Bedford, MA, USA); 3-(4,5-dimethylthiazol-2-yl)-2,5-diphenyltetrazolium bromide (MTT) was obtained from Sigma (St. Louis, MO, USA); Cell culture media consisted of Dulbecco’s Modified Eagles Essential Medium (DMEM, Invitrogen, Carlsbad, CA, USA) to which 10 % fetal bovine serum (FBS, Sigma, St. Louis, MO, USA) and 1 % pen/strep (P/S, Sigma, St. Louis, MO, USA); Dimethylsulfoxide (DMSO) were purchased from ATCC (Rockville, MD, USA); 48 and 96-well cell culture clusters and other plastic disposables were purchased from Corning Inc. (Corning, NY, USA). Transforming growth factor beta 1 (TGF-β1) (Sigma, St. Louis, MO, USA) was added to some cultures to transform a fraction of the MRC-5 fibroblasts into cancer-associated fibroblasts (CAFs).

Type I collagen was selected as the 3-D scaffold for cell culture because it resembles the extracellular matrix milieu of invasive breast carcinoma better than other polymers. Collagen solutions were diluted from 10 mg/ml and neutralized with DMEM medium and 1 N sodium hydroxide to 2.0 mg/ml. Cells were introduced to a particular sample at this time, before matrix polymerization. 600 μl volumes of collagen solution were dispensed to a 48-well plate and incubated at 37 °C as the collagen polymerized over 30 min. After polymerization, 300 μl of complete DMEM was applied to the top of the gelled samples. With or without cells, the total fluid volume added to each sample was 600 μl so that matrix stiffness was not influenced by medium volume.

Co-cultures were constructed by mixing 60,000 MCF-7 epithelial cells and 200,000 MRC-5 fibroblast cells (1:3 ratio) into 600 μl of collagen matrix to mimic conditions of residual disease in the post-surgical human breast. Many researchers have used the 3/1 ratio breast cancer cell lines (i.e. MCF-7) to fibroblast (i.e. MRC-5) to examine breast cancer. However, we wanted to examine the post-surgical environment, thus looking at the influence of a microenivironment in a post-surgical region [17–20]. Values were chosen after evaluating the sustainability and proliferation responses of cell samples. At higher initial cell densities, MCF-7 cells proliferated continuously and overtook samples. At lower initial cell densities, MCF-7 cells had difficulty sustaining growth. The initial density of MRC-5 cells was optimized in our previous work for the sample volume. The same initial cell densities were seeded in samples where each cell type was cultured independently. Cultures were maintained for 5 days and the medium was changed every 2–3 days.

### Sample irradiation

Samples were irradiated by placing them in a 6 MV photon field generated by a linear accelerator (Varian Medical System, Palo Alto, CA, USA) as described previously [4]. Briefly, a uniform dose was delivered with the gantry set to 0° and a 100 cm source-to-axis distance. An 8 × 8 cm^2^ field size was selected to ensure cell-culture samples were uniformly irradiated. Dosimetric-quality solid water was placed above and below the well plates to provide proper build-up dose. All samples were taken from the incubator and placed in a thermally isolating container for transportation to and from the clinic. Samples were removed from the container, exposed to ionizing radiation (IR) at room temperature in a single dose, and returned to the incubator within 30 min. The standard clinical dose fraction for breast treatment of 180 cGy was delivered to samples at a rate of 400 cGy/min. Other samples were exposed at the same dose rate but at a reduced fraction size of 90 cGy or an accelerated fraction size of 360 cGy. All sample irradiations were delivered in 5 fractions, once each 24 h over 5 consecutive days, so the total dose was 450, 900, or 1800 cGy. Dose delivery verification was conducted following clinical standards.

### MTT assays

In order to approximate the metabolic activity of irradiated cells, the 3-(4, 5-dimethylthiazol-2-yl)-2, 5-diphenyltetrazolium bromide (MTT) assay was used. This sensitive, quantitative and reliable assay measures the conversion of MTT substrate produced in cells into a formazan salt by cellular dehydrogenase [21]. MTT assesses cellular metabolism based on the ability of the mitochondrial succinate-tetrazolium reductase system to convert the MTT into formazan. Viable cells reduce the water-soluble yellow-colored MTT to a water-insoluble purple colored formazan product in proportion to their metabolic rate. The amount of colored formazan product formed, as determined spectrophotometrically after dissolving the formazan crystals in DMSO, is proportional to the net metabolic activity of cells with functioning mitochondria in the sample. The MTT assay is a common tool used in radiobiological studies to assess metabolic activity [17]. We applied it to *in-vitro* cell cultures for the assessment of cellular metabolism, survival and proliferation as described below.

We were unable to reliably use MTT assays in 3-D cultures, so we performed 2-D culture studies to measure metabolic activity and 3-D cultures to measure cell proliferation. Then we compared them to establish a calibration in which either measurement described cell proliferation.

Cells harvested from culture flasks by trypsinization were resuspended in new phenol free media, plated at different cell densities in 96-well culture plates, and incubated at 37 °C in 5 % CO_2_. The cell medium was replaced every 2–3 days. The cell monolayer is treated for 4 h with MTT dissolved in PBS (concentration 5 mg*/*ml) at 37 °C and 5 % CO_2_. After incubation, the MTT solution is removed and 100 *μ*l DMSO is added to each well of the 96-well plate to dissolve the formazan crystals. Plates were shaken for 10 min to ensure adequate solubilization. Assessment of metabolic activity was recorded as relative colorimetric changes measured at 570 nm. Samples were read in an Emax reader (Molecular Devices, Wokingham, UK) with Softmax PRO version 4.3 software. Control-sample wells for absorbance readings contained no cells or medium but MTT solution was added as per experimental wells on the plate. They were removed after incubation and DMSO was added. All experiments were performed at least 3 times and the results were averaged.

### Immunofluorescence staining (IF)

MRC-5 and MCF-7 cells were mixed in type I collagen at a final collagen concentration of 2 mg/ml to form 3-D co-cultures. Samples were maintained in DMEM containing 10 % FBS and 1 % P/S at 37 °C in a humidified atmosphere containing 5 % CO_2_. To quantify the cell growth in collagen gel following mechanical testing, each sample was washed with Phosphate-Buffered Saline (PBS, Lonza, Wackersville, MD, USA) before being fixed with 4 % paraformaldehyde at 4 °C overnight. Samples were then washed and permeabilized in 0.02 % Triton X-100 in PBS for 15 mins followed by 5 % non-fat milk blocking for 2 h. Gels were then incubated with primary monoclonal α-SMA antibody, consisting of Bovine Serum Albumin (BSA, Sigma), PBS and Tween 20 (T, Sigma), (1:100 in 1 % BSA/PBS/T) overnight at 4 °C and secondary antibody (1:200 in 1 % BSA/PBS/T) at room temperature for 2 h. Cell nuclei were stained with TO-PRO 3 (1:1000 in PBS) for 20 mins. Sections were mounted between two glass coverslips with anti-photobleaching reagent.

### Confocal microscopy

Immunofluorescence-stained 3-D cultures were examined with a Leica SP2 laser scanning confocal microscope (Leica, Heidelberg, Germany) with Hg lamp, helium/neon laser, and the associated software (Leica Confocal Software Version 2.00). A 488 nm excitation wavelength beam was applied for Fluorescein isothiocyanate (FITC) mapping, and a 633 nm excitation wavelength beam was used to find cell nuclei via TO-PRO 3 mapping. For each sample, a 20X objective captured a series of images along the z-coordinate (sample depth) at 3–5 μm depth increments. We used a confocal microscopy system (Microradiance; Bio-Rad, Philadelphia, PA, USA) consisting of a 25-Mw argon ion laser emitting at 488 and 514 nm and a 1-Mw green helium–neon laser emitting at 546 nm attached to a BX-50 microscope (Olympus Imaging America, Inc., Center Valley, PA, USA).

### Automated area quantification for cell counting

**Fig. 7:**
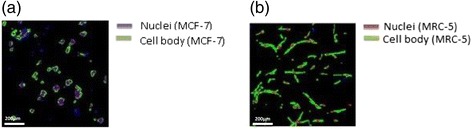
Immunofluorescence staining of epithelial and fibroblast cell lines. Cell staining was done with α-SMA **a** MCF-7 cells in monoculture clustered into small spheroids. **b** MRC-5 cells appear distinct from each other with long-tail cell bodies and clearly identified nuclei. Scale bar = 200 μm

MCF-7 cells were found to aggregate in clusters as cells proliferated in the 3-D collagen matrix. Their cellular morphology was obviously different from the MRC-5 cells, and so the two were easily distinguished. MCF-7 cells present relatively rounded and less spread morphologies while the MRC-5 cells appeared as long-tailed bodies expressing alpha-smooth muscle actin (α-SMA) with distinct nuclei, as shown in Fig. 7. These imaging techniques were used to quantify proliferation rates for each cell type.

The numbers and areas of cells occupied by each type were counted using automated segmentation software developed for the project was applied to digital microscope images (Qayyum and Kwak). To differentiate MRC-5 and MCF-7 cells in co-culture samples, both TO-PRO3 red and FITC green fluorescent (GF) images were recorded and merged. In the MCF-7 cell growth assay, a nucleus size exclusion strategy was applied during the cell counting procedure. MRC-5 cells displayed a more elongated nucleus than MCF-7 cells. We collected the circular regions and then analyzed the MCF-7 cell number and area. We calibrated the automated image counting method with a hand counting method to validate accuracy. Coupling information from IF image analysis with MTT assays, we estimated viable cell numbers during the five-day experiment to estimate cell proliferation rates.

### Mechanical testing

The protocol for mechanical testing is detailed in our previous work [4]. Briefly, samples were tested using a TA.XT Plus Texture Analyzer System with a 1 kg load cell (Texture Technologies Corp., Scarsdale, NY, USA) and a compressive indentation procedure within 1 h following delivery of IR. The fluid medium was removed from the top of the sample before testing with a 5-mm-diameter spherical indenter. The indenter was pressed into the sample surface under quasi-static conditions (indenter velocity = 10^−1^ mm/min) and to a depth of 2.5 mm. Force (F) and displacement (d) were recorded by the instrument to estimate sample stiffness. Force-displacement plots show how force sensed by the indenter varied with indenter depth during loading and unloading phases. Stiffness k is defined as the slope F(d)/d near d = 0.

### Statistical analysis

Analysis of variance (ANOVA) was performed using R software to determine the significance of differences at α = 0.05 level of significance. Observed differences among mean sample groups were evaluated with post-hoc analysis, including Tukey HSD. This method was similar to earlier publication [4].

## References

[1] Elenbaas B, Weinberg RA (2001). Heterotypic signaling between epithelial tumor cells and fibroblasts in carcinoma formation. Exp Cell Res.

[2] Butcher DT, Alliston T, Weaver VM (2009). A tense situation: forcing tumour progression. Nat Rev Cancer.

[3] Pinto MP, Badtke MM, Dudevoir ML, Harrell JC, Jacobsen BM, Horwitz KB (2010). Vascular endothelial growth factor secreted by activated stroma enhances angiogenesis and hormone-independent growth of estrogen receptor-positive breast cancer. Cancer Res.

[4] Qayyum MA, Insana MF (2012). Stromal responses to fractionated radiotherapy. Int J Radiat Biol.

[5] Alberts B (2002). Molecular biology of the cell.

[6] Bromley R, Davey R, Oliver L, Harvie R, Baldock C (2006). A preliminary investigation of cell growth after irradiation using a modulated x-ray intensity pattern. Phys Med Biol.

[7] Abram WP, Clarke J, Mcaleer JJA, Graham JD, Riddle P, Goodman S (2008). The UK Standardisation of Breast Radiotherapy (START) trial a of radiotherapy hypofractionation for treatment of early breast cancer: a randomised trial. Lancet Oncol.

[8] Gouk SS, Lim TM, Teoh SH, Sun WQ (2008). Alterations of human acellular tissue matrix by gamma irradiation: histology, biomechanical property, stability, in vitro cell repopulation, and remodeling. J Biomed Mater Res B Appl Biomater.

[9] Nguyen TD, Maquart FX, Monboisse JC (2005). Ionizing radiations and collagen metabolism: from oxygen free radicals to radio-induced late fibrosis. Radiat Phys Chem.

[10] Rodemann HP, Blaese MA (2007). Responses of normal cells to ionizing radiation. Semin Radiat Oncol.

[11] Morishima Y, Nomura A, Uchida Y, Noguchi Y, Sakamoto T, Ishii Y (2001). Triggering the induction of myofibroblast and fibrogenesis by airway epithelial shedding. Am J Respir Cell Mol Biol.

[12] Olumi AF, Grossfeld GD, Hayward SW, Carroll PR, Tlsty TD, Cunha GR (1999). Carcinoma-associated fibroblasts direct tumor progression of initiated human prostatic epithelium. Cancer Res.

[13] Desmouliere A, Guyot C, Gabbiani C (2004). The stroma reaction myofibroblast: a key player in the control of tumor cell behavior. Int J Dev Biol.

[14] Levental KR, Yu H, Kass L, Lakins JN, Egeblad M, Erler JT (2009). Matrix crosslinking forces tumor progression by enhancing integrin signaling. Cell.

[15] Owen JR, Ashton A, Bliss JM, Homewood J, Harper C, Hanson J (2006). Effect of radiotherapy fraction size on tumour control in patients with early-stage breast cancer after local tumour excision: long-term results of a randomised trial. Lancet Oncol.

[16] Prosnitz LR, Horton J, Wallner PE (2009). Accelerated partial breast irradiation: caution and concern from an ASTRO task force. Int J Radiat Oncol Biol Phys.

[17] Rossi L, Reverberi D, Podesta G, Lastraioli S, Corvo R (2000). Co-culture with human fibroblasts increases the radiosensitivity of MCF-7 mammary carcinoma cells in collagen gels. Int J Cancer.

[18] Sadlonova A, Novak Z, Johnson M, Bowe D, Gault S, Page G (2005). Breast fibroblasts modulate epithelial cell proliferation in three-dimensional in vitro co-culture. Breast Cancer Res.

[19] Krause S, Maffini M, Soto A, Sonnenschein C (2010). The microenvironment determines the breast cancer cells’ phenotype: organization of MCF7 cells in 3D cultures. BMC Cancer.

[20] Seslar SP, Nakamura T, Byers SW (1993). Regulation of fibroblast hepatocyte growth factor/scatter factor expression by human breast carcinoma cell lines and peptide growth factors. Cancer Res.

[21] Slater TF (1963). Studies on a succinate-neotetrazolium reductase system of rat liver II. Points of coupling with the respiratory chain. Biochimica et Biophysica Acta.

